# Supplementary material for: Hippocampal Place Cells with NMDARs Do Not Require Excitation and Inhibition to Be Reciprocally Tuned
Source: bioRxiv. 2026 Apr 29:2026.04.27.721108. Preprint. [Version 1] doi: 10.64898/2026.04.27.721108 (PMC13142514; doi:10.64898/2026.04.27.721108)
Supplement: Supplement 1 [file NIHPP2026.04.27.721108v1-supplement-1.pdf]

bioRxiv preprint doi: https://doi.org/10.64898/2026.04.27.721108; this version posted April 29, 2026. The copyright holder for this preprint (which was not certified by peer review) is the author/funder, who has granted bioRxiv a license to display the preprint in perpetuity. It is made available under aCC-BY 4.0 International license.

Supplementary Information

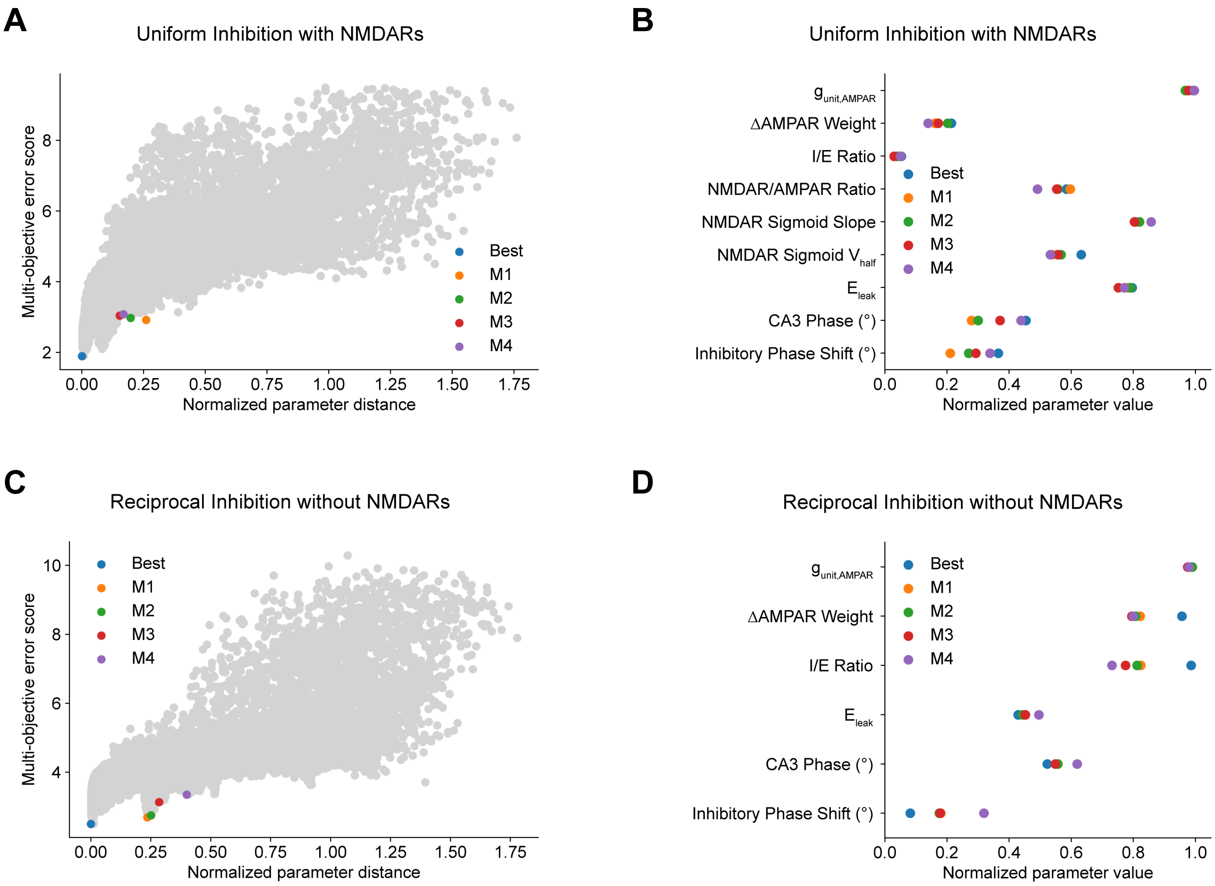

Supplementary Figure S1.

A, During parameter optimization, CA1 place cell model variants with different parameters were evaluated against criterion based on experimental targets. A family of models were chosen that performed similarly across optimization criterion, but exhibited diversity in their parameter values. Shown are model variants of the model configuration with uniform inhibition and with NMDARs. **B,** Parameter values are shown for the five model variants labeled in A. **C-D,** Same as A-B for the model configuration with reciprocal inhibition and without NMDARs.

bioRxiv preprint doi: https://doi.org/10.64898/2026.04.27.721108; this version posted April 29, 2026. The copyright holder for this preprint (which was not certified by peer review) is the author/funder, who has granted bioRxiv a license to display the preprint in perpetuity. It is made available under aCC-BY 4.0 International license.

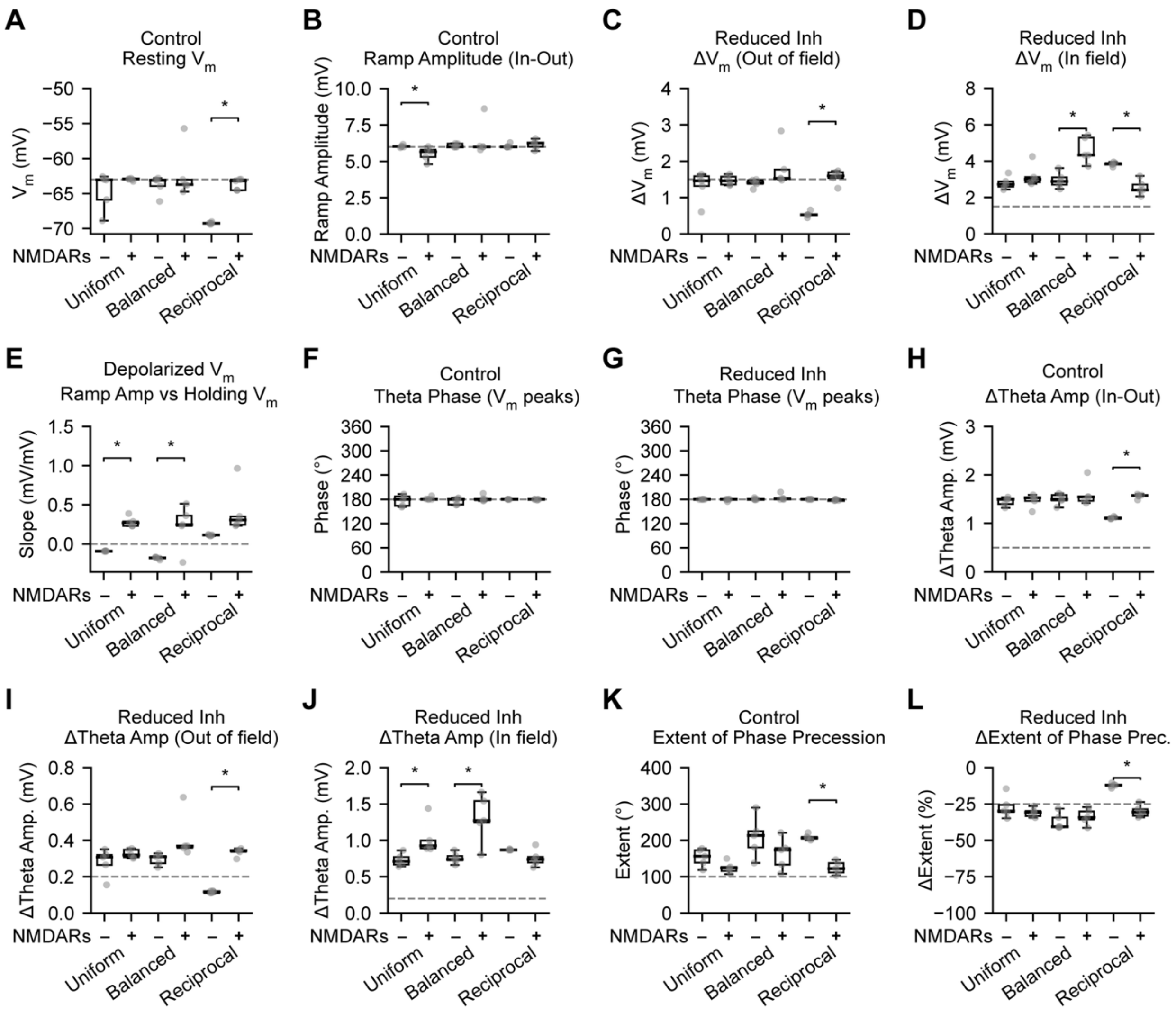

Supplementary Figure S2.

A-L, For each CA1 place cell model configuration, feature values were measured and compared against experimental targets for five variants of each model with different alternative parameters. Data is displayed as box-and-whisker plots with individual data points, group medians and inter-quartile ranges indicated.

bioRxiv preprint doi: https://doi.org/10.64898/2026.04.27.721108; this version posted April 29, 2026. The copyright holder for this preprint (which was not certified by peer review) is the author/funder, who has granted bioRxiv a license to display the preprint in perpetuity. It is made available under aCC-BY 4.0 International license.

		Simple Model without NMDARs			Simple Model with NMDARs		
**Parameter**	**Bounds**	Uniform	Balanced	Reciprocal	Uniform	Balanced	Reciprocal
$g_{unit,AMPAR}$ (a.u.)	1.E-07 – 1.E-04	9.8670E-05	1.3813E-05	9.7497E-05	9.9413E-05	8.7904E-05	9.0890E-05
∆AMPAR Weight (a.u.)	1 – 10	8.9372	9.2579	8.0245	2.6187	3.6835	2.4229
I/E Ratio (a.u.)	0.5 – 10	8.9185	0.5098	9.4067	0.8612	1.0129	0.9276
NMDAR/AMPAR Ratio (a.u.)	0 – 10	N/A	N/A	N/A	0.3372	0.5623	0.2755
NMDAR Sigmoid Slope (a.u.)	0.001 – 1	N/A	N/A	N/A	0.3356	0.3013	0.3148
NMDAR Sigmoid V_{half} (mV)	-80 – -20	N/A	N/A	N/A	-45.8214	-45.5522	-45.2547
E_{leak} (mV)	-80 – -60	-72.0105	-65.5976	-71.3614	-64.1191	-63.7985	-64.0118
CA3 Phase (°)	100 – 180	161.6478	135.2596	165.0183	133.4073	134.4106	135.7249
Inhibitory Phase Shift (°) (Relative to CA3)	-30 – 30	-8.1176	-26.6096	-6.3470	-10.5210	-7.9393	-8.5534
Number of connections from positively tuned interneurons	N/A	250	291	232	250	291	232
Number of connections from negatively tuned interneurons	N/A	238	265	218	238	265	218

Supplementary Table S1.
Bounds and optimized parameters are shown for six CA1 place cell model configurations.
